# Ameliorative effects of sildenafil against carbon tetrachloride induced hepatic fibrosis in rat model through downregulation of osteopontin gene expression

**DOI:** 10.1038/s41598-024-67305-1

**Published:** 2024-07-23

**Authors:** Hend Elsayed Nasr, Ahmed Medhat Hegazy, Noha Osama El-Shaer, Rabab Shaban El-shafey, Salwa A. Elgendy, Heba A. Elnoury, Walaa Bayoumie El Gazzar, Lina Abdelhady Mohammed

**Affiliations:** 1 Department of Medical Biochemistery and Molecular Biology, Faculty of Medicine, Benha University, Benha, 13518 Egypt; 2 Department of Forensic Medicine and Toxicology, Faculty of Veterinary Medicine, Benha University, Moshtohor, Toukh, 13736 Qalyubia Egypt; 3 Department of Physiology, Faculty of Medicine, Benha University, Benha, 13518 Egypt; 4 Department of Forensic Medicine and Clinical Toxicology, Faculty of Medicine, Benha University, Benha, 13518 Egypt; 5 Deparment of Pharmacology, Faculty of Medicine, Benha University, Benha, 13518 Egypt

**Keywords:** Carbon tetrachloride, Hepatoprotective, Osteopontin, Oxidative markers, Sildenafil, Biochemistry, Drug discovery, Physiology, Biomarkers, Medical research

## Abstract

The liver carries out many essential tasks, such as synthesising cholesterol, controlling the body’s storage of glycogen, and detoxifying metabolites, in addition to performing, and regulating homeostasis. Hepatic fibrosis is a pathological state characterized by over accumulation of extracellular matrix (ECM) including collagen fibers. Sildenafil (a selective inhibitor of type 5 phosphodiesterase) has anti-inflammatory, antioxidant and anti-apoptotic properties. It is commonly used to treat erectile dysfunction in male. The purpose of the current investigation was to evaluate sildenafil’s hepatoprotective potential against liver fibrosis in rats that was caused by carbon tetrachloride (CCl_4_). Liver enzymes and oxidative markers as well as profibrotic genes were determined. The findings showed that sildenafil alleviates the hepatic dysfunctions caused by CCl_4_ by restoring normal levels of ALT, AST, and GGT as well as by restoring the antioxidant status demonstrated by increased glutathione (GSH), and catalase. In addition, a significantly down-regulated the mRNA expressions of profibrotic genes [collagen-1α, IL-1β, osteopontin (OPN), and transforming growth factor-β (TGF-β)]. Additionally, sildenafil lessens the periportal fibrosis between hepatic lobules, congestion and dilatation in the central vein, and the inflammatory cell infiltrations. As a result, it is hypothesized that sildenafil may be helpful in the management of hepatotoxicity brought on by CCl_4_ through suppressing OPN.

## Introduction

The liver is a major organ in the body as it carries out a multitude of essential tasks, such as metabolite detoxification, cholesterol synthesis, and glycogen storage regulation, as well as body performance and homeostasis regulation^1^. Owing to its various metabolic functions, the liver is vulnerable to several illnesses. The major health problem is liver fibrosis which may lead to liver cirrhosis and liver cancer. A multifactorial process is driving chronic liver disease to cirrhosis^2^. Hepatic fibrosis is a pathological state characterized by over accumulation of ECM including collagen fibers; it is a ‘mis-repair’ occurs in response to an inflammatory reaction following chronic liver injury that resulting in activating hepatic stellate cells (HSCs) that considered the main source of myofibroblasts of the fibrotic liver^3^. These cells lead to degradation of the normal ECM of the liver, release of cytokines, and organ contraction^4^. Liver fibrosis may be progressive and leads to cirrhosis. If the liver is not at the stage of advanced cirrhosis, the fibrotic response-causing agent can aid in the regression of fibrosis. Some of these cellular signals include morphogens, growth factors, and cytokines^5^. Osteopontin (OPN) is a highly phosphorylated glycoprotein, it was first identified in malignant cells; it was found in many tissues such as bile, milk, saliva, dentin layer of the tooth, brain, bone marrow, kidney, and liver^6^. It considered as a significant chemical attractant for T cells and macrophages during inflammation^7^. Data reported that OPN gene is associated with the progression and pathogenesis of cancer, autoimmune, neurodegenerative, cardiovascular and renal diseases^8^. Increased hepatic OPN expression was accompanied with fibrosis in patients with HBV and HCV infection, schistosomiasis mansoni, alcoholic liver diseases and as a marker for the conversion of liver fibrosis to cirrhosis. It also served as a diagnostic marker of portal hypertension and HCC^9^. OPN exists in a secreted form that mediates cell adhesion, migration and survival, and an intracellular non-secreted form. During hepatic fibrogenesis, activation of quiescent HSCs is promoted by OPN then subsequently increases the expression and secretion of collagen-1^10^.

Sildenafil, a phosphodiesterase-5 inhibitor, has anti-apoptotic, antioxidant and anti-inflammatory properties, and improve the immune system function; it decreases oxidative stress in brain and kidneys of rats with cisplatin or ischemia that induced acute kidney injury (AKI) and diabetic nephropathy^11^. The drug ameliorated smoke-induced lung inflammation, and inflammatory demyelination in rats^12^. Thus, the purpose of this study was to assess sildenafil’s hepatoprotective effects against liver fibrosis in wistar rats caused by CCl_4_.

## Materials and methods

### Ethical approval

The Institutional Animal Ethics Committee at Benha University in Egypt provided instructions and procedures that were followed during the conduct of this experiment. The protocol number for approval is BUFVTM 09-11-22. All methods were performed in accordance with the relevant guidelines and regulations. The study is reported in accordance with ARRIVE guidelines.

### Chemicals

Sildenafil (Viagra^®^) (100 mg/tablet) was purchased from Pfizer Co., Egypt. The carbon tetrachloride (CCl_4_) was purchased from NICE Co., India.

### Experimental animals

At six weeks of age and weighing between 125 and 145 g, forty adult male Wistar rats were procured from the animal house of the National Research Centre in Egypt (Dokky, Giza, Egypt). The rats were provided with free, clean water, a balanced diet, and a clean environment in which to live. The rats were housed in a natural 12 h light/dark cycle with an ambient temperature of 23 ± 3 °C. Rats were burned in an incinerator following the completion of the experiment and sample collection.

### Experimental design

Four equal groups of ten rats each were randomly assigned to the experimental rats. The group 1 (G1) was preserved as a normal control, and the group 2 (G2) was administered orally sildenafil dissolved in saline by stomach tube at a dose of 20 mg/kg b.wt.^13^. daily for 4 weeks. The group 3 (G3) (intoxicated group) was administered intraperitoneal (I/P) CCl_4_ (30% in olive oil) at a dose of 3 mg/kg b.wt ^14^. twice a week for 4 weeks. Group 4 (G4), which received co-treatment, received sildenafil via stomach tube at a dose of 20 mg/kg b.wt. daily and CCl_4_ I/P at a dose of 3 mg/kg b.wt. twice a week for 4 weeks. Throughout the experiment, all groups’ clinical symptoms were noted. At the end of the experiment, the experimental rats were euthanized using isoflurane inhalation. Blood for serum samples was collected from the abdominal aorta, and liver specimens were collected from all rats in each group at the end of the fourth week of the experiment for evaluation of liver function, oxidative markers, gene expression, histopathological changes, and immunohistochemically markers.

### Preparation of liver homogenate

The liver tissue homogenate was made in compliance with Farid and Hegazy^15^. The total protein and the oxidative indicators [catalase (CAT), reduced glutathione (GSH), and lipid peroxidation by-products (MDA) level] were measured in the obtained supernatant.

### Assay methods

#### Liver function tests

The activity of alanine aminotransferase (ALT)^16^, aspartate aminotransferase (AST)^16^, and gamma glutamyltransferase (GGT)^17^ were performed.

#### Oxidative markers in liver homogenate

The levels of MDA^18^, GSH^19^, CAT^20^, and the total proteins content^21^ were performed in the liver homogenates.

#### Hepatic mRNA gene expression

The mRNA expression of hepatic collagen-1α, interleukin-1β (IL-1β), osteopontin (OPN), and TGF-β genes were analyzed using a 7300 real-time PCR system (PCR) (Applied Biosystems, Foster City, CA, USA). The expression of hepatic collagen-1α, IL-1β, OPN, and TGF-β cytokines and β-actin (as a housekeeping gene) were analyzed with sense and antisense primers according to a previously published method^22^ used the primers sets (Table 1). The changes in gene expression were estimated from the obtained cycle threshold (Ct) values provided by the real-time PCR equipment using the comparative Ct method to a reference (β-actin, housekeeping gene)^23^.

**Table 1:** The primer sets of the assessed genes.

Genes	Forward primer (sense)	Reverse primer (anti-sense)	Gen Bank ID
Collagen-1α	5′- TCTCAAGATGGTGGCCGTTA-3′	5′-ATCTGCTGGCTCAGGCTCTT-3′	NM_053304.1
IL-1β	5′-CAC CTC TCA AGC AGA GCA CAG-3′	5′-GGG TTC CAT GGT GAA GTC AAC-3′	NM_031512
Osteopontin	5′- CAGTGATTTGCTTTTGCCTGTTTG -3′	5′- GGTCTCATCAGACTCATCCGAATG-3′	XM_008769996
TGF-β	5′-TGACATGAACCGACCCTTCC-3′	5′-CCAGGCTCCAAATGTAGGGG-3′	NM_021578
β-actin	5′- AAGATCCTGACCGAGCGTGG-3′	5′- CAGCACTGTGTTGGCATAGAGG-3′	NM_031144

#### Histopathological examinations

Tissue specimens from liver of each rat were collected immediately after euthanized, rinsed with isotonic saline, and fixed in 10% neutral buffered formalin and dehydrated by serial ethanol solutions (70, 95, and 100%, respectively). Paraffin-embedded tissue sections (~ 5 μm thickness), and stained with hematoxylin and eosin (H&E) and with Masson’s trichrome^24^. For a histopathological assessment, the slides were inspected using a light microscope (Olympus BX50, Japan).

#### Immunohistochemistry staining

The liver slices (5 µm) were adhered to glass microscope slides with positive charge. Sections deparaffinized and then rehydrated with decreasing alcohol concentration. For thirty minutes, a microwave was used to heat-induced antigen retrieval using a citrate buffer solution. After allowing the sections to cool at room temperature, nonspecific binding was stopped for 20 min at that temperature using regular goat serum. Incubation with the diluted primary antibodies [alpha-smooth muscle actin (α-SMA) Monoclonal Antibody, Catalog # 14-9760-82, Thermo Fisher Scientific, Manor Park, United Kingdom] at a dilution rate of 1:200 for 60 min at 37 ℃. After that, sections were exposed to a peroxidase suppressor for 15 min at room temperature in order to inhibit the activity of endogenous peroxidase. Subsequently, the DAB chromogen mixture was applied to each slide after they had been incubated for one hour at room temperature with the secondary antibody (goat anti-rat peroxidase conjugated streptavidin). PBS was used for washing and drying after each step. To show the nuclei, sections were counter-stained with Meyer’s hematoxylin. A dark brown response indicated good immuno-expression. Except for adding the primary antibodies, a negative control underwent the previously described procedures^25^.

#### Image analysis

Using an Olympus CX51 microscope, the percentage of the area (area %) covered by the blue color in the Masson’s trichrome staining or immunostaining area for α-SMA was examined, and photos were captured with an Olympus digital camera (E-620, United States). Digital image analysis was used to evaluate and quantify the images using the computer programme Scion Image Beta 4.03 (Scion Corporation, USA).

### Statistical analysis

The information was shown as mean ± standard deviation (SD). ANOVA was used to determine the significance of mean differences, and Duncan’s multiple range test was then performed using SPSS for Windows (version 20.0; SPSS Inc., Chicago, Ill.). The difference of means at *P* < 0.05 is considered significant.

### Consent to participate

Acceptance of participation, each individual participant in this study provided both oral and written informed permission.

## Results

No any symptoms or rat died in any of the experimental groups over the course of the experiment.

### Liver function tests

After the fourth week of treatment, the CCl_4_-treated group’s levels of ALT, AST, and GGT considerably increased in comparison to the other experimental groups. However, following the fourth week of the trial, ALT, AST, and GGT levels were mitigated in the group that received sildenafil and CCl_4_ (Table 2).

**Table 2:** Effect of sildenafil against carbon tetrachloride (CCl_4_) on liver function test in various gatherings of the experiment after 4 weeks of treatment (mean ± standard deviation), (*n* = 10).

	Control	Sildenafil	CCl_4_	Sildenafil+CCl_4_
ALT (U/L)	27.469 ± 3.322^c^	27.697 ± 3.836^c^	70.172 ± 5.416^a^	37.316 ± 3.704^b^
AST (U/L)	76.892 ± 4.691^c^	77.424 ± 4.519^c^	159.701 ± 4.954^a^	108.549 ± 7.451^b^
GGT (U/L)	1.607 ± 0.597^c^	1.964 ± 0.329^c^	8.855 ± 0.999^a^	3.221 ± 0.837^b^

Means with different superscripts in the same row are significantly different at *P* < 0.05.

### Changes in the liver oxidative markers

When compared to the other experimental groups, the CCl_4_-treated group’s liver tissue exhibited considerably lower GSH contents and lower CAT activities. However, following the fourth week of the trial, the group that received both sildenafil and CCl_4_ co-treatment exhibited alleviation of these activities. When compared to the other experimental groups, the CCl_4_-treated group's levels of MDA, a marker of LPO in liver tissue, were also significantly higher; however, after the fourth week of the experiment, the group that received both sildenafil and CCl_4_ co-treatment showed mitigation of MDA levels (Table 3).

**Table 3:** Effect of sildenafil against carbon tetrachloride (CCl_4_) on oxidative markers in the liver homogenates of various gatherings of the experiment after 4 weeks of treatment (mean ± standard deviation), (*n* = 10).

	Control	Sildenafil	CCl_4_	Sildenafil+CCl_4_
MDA (nmol/mg protein)	6.329 ± 0.844^c^	7.149 ± 1.061^c^	31.198 ± 1.985^a^	14.831 ± 2.411^b^
GSH (µmol/mg protein)	20.905 ± 2.541^a^	19.076 ± 3.107^a^	4.652 ± 1.146^c^	13.613 ± 1.303^b^
CAT (unit/mg protein)	5.969 ± 0.673^a^	5.675 ± 0.877^a^	1.359 ± 0.475^c^	3.435 ± 0.642^b^

Means with different superscripts in the same row are significantly different at *P* < 0.05.

### Collagen-1α, IL-1β, OPN, and TGF-β mRNA expression in liver tissue

**Figure 1:**
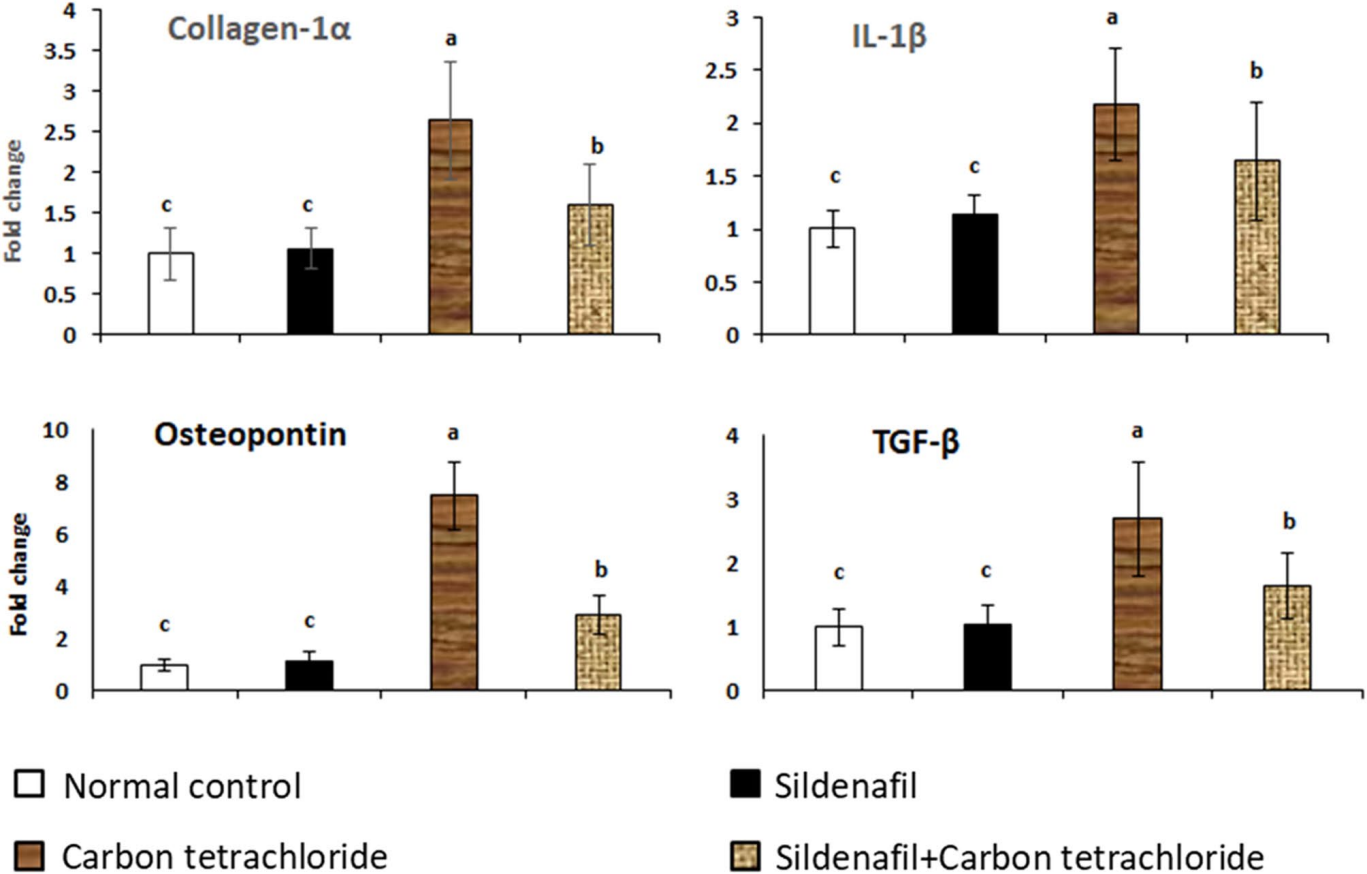
mRNA expression of hepatic collagen-1α, IL-1β, OPN, and TGF-β. Total RNA was prepared from hepatic tissues of rats treated with sildenafil, CCl_4_, sildenafil against CCl_4_, and control on 4 weeks after treatments. Real-time PCR was evaluated the expression levels. *P* ˂ 0.05 compared with control values. Bars represent means ± SDM. (*n* = 10).

Figure 1 shows the expression of mRNA genes for liver collagen-1α, IL-1β, OPN, and TGF-β in the control group and groups treated with sildenafil, CCl_4_, and sildenafil+CCl_4_ after four weeks of treatment. The liver mRNA of the rats treated with CCl_4_ showed a significant elevation of collagen-1α, IL-1β, OPN, and TGF-β. Conversely, there was a noticeable downregulation of these genes in the group that received sildenafil and CCl_4_ together.

### Histopathological assessment of liver tissue

**Figure 2:**
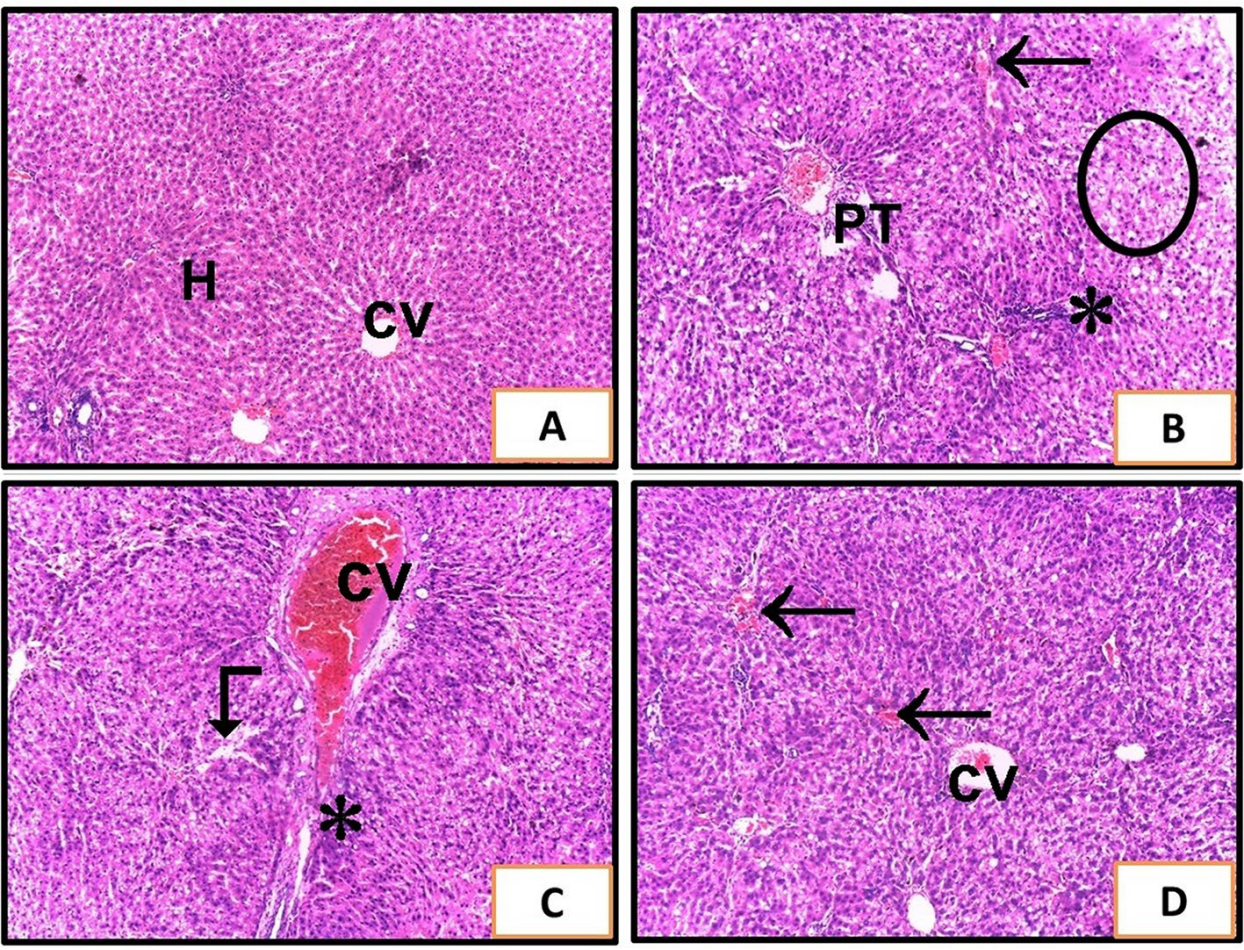
Liver sections of rats from different experimental groups. (**A**) The control group (G1) and (G2) received sildenafil and showed a normal histological features of the liver with no abnormalities. Cords of hepatocytes (H) radiating from the central vein (CV). (**B**,**C**) show liver of G3 rats that received CCl_4_. (**B**) Shows sever degenerative changes with distorted histological architecture, vacuolization (circle), congested portal tract vasculature (PT), large inflammatory cells infiltrates (asterisk), and congested sinusoids (arrow). (**C**) Shows Sever congested central vein (cv), dilated sinusoids (angled arrow), and large inflammatory cells infiltrates (asterisk). (**D**) G4 received sildenafil and CCl_4_ and showed improvement of hepatic architecture with congestions between some hepatocytes (H&E ×100).

Examination of liver sections stained with H and E revealed that CCl_4_ treated rats showed sever degenerative changes with distorted histological architecture, vacuolization (circle), sever congested central vein (CV), congested portal tract vasculature (PT), large inflammatory cells infiltrates (asterisk), some congested sinusoids (arrow), and other dilated sinusoids (angled arrow) were seen (Fig. 2B, C). Treatment of rats with sildenafil and CCl_4_ showed improvement of the hepatic architecture but mild congestions between the hepatocytes were still noticed (Fig. 2D). The hepatic tissues of rats that administered sildenafil appeared to be normal (Fig. 2A).

### Masson’s trichrome stain examination

**Figure 3:**
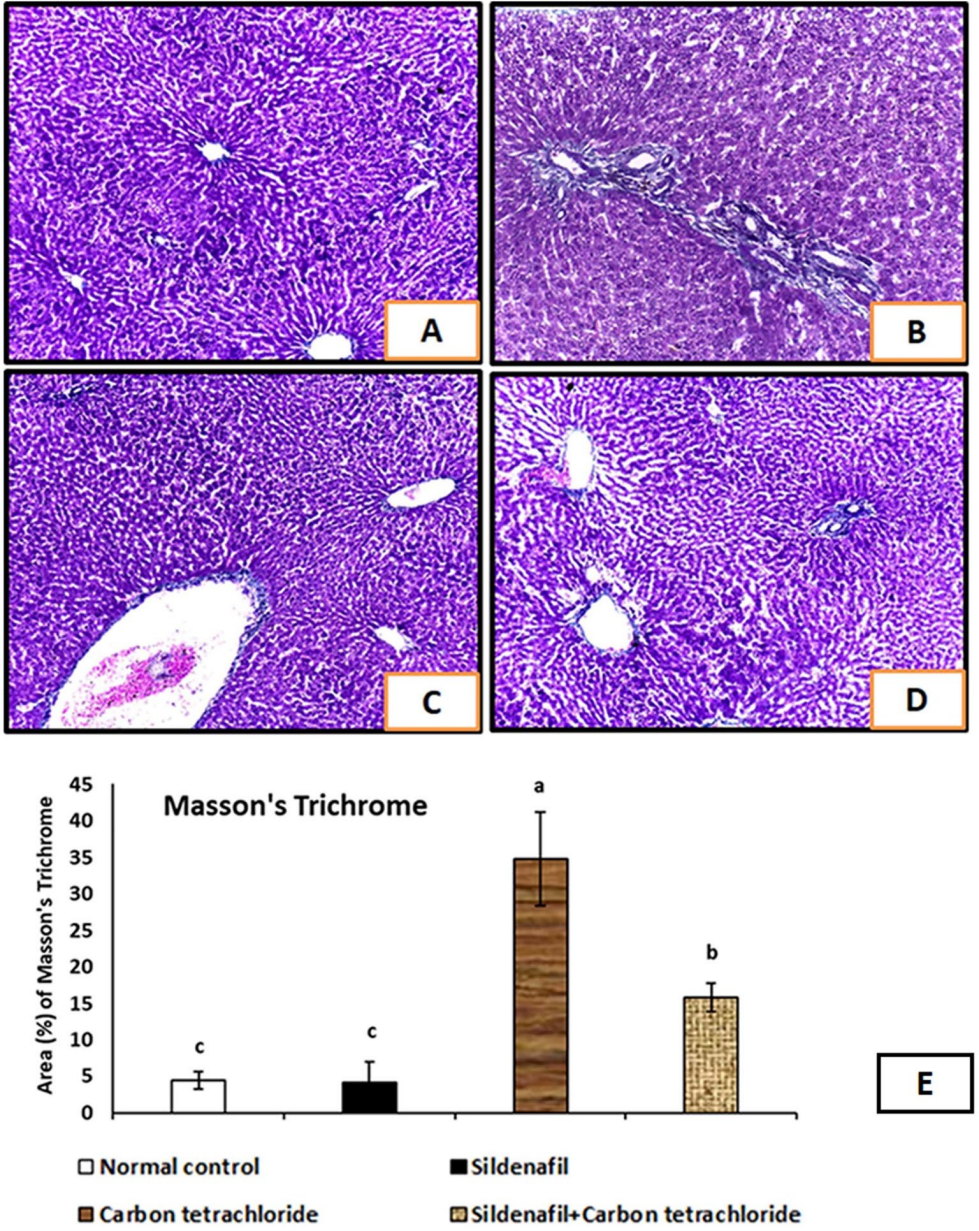
Liver sections of rats from different experimental groups stained with Masson’s trichrome. (**A**) The control group (G1) and (G2) received sildenafil and showed minimal collagen expression just around the central veins. (**B**,**C**) Show liver of G3 rats that received CCl_4_; showed increased collagen fibers deposition in the portal area and around the central veins. (**D**) G4 received sildenafil and CCl_4_ and showed reduced collagen deposition compared to G3 group. (Masson’s trichrome ×100). (**E**) Show digital morphometric study of area (%) of Masson’s trichrome of liver of experimental rats. Data were used to estimate the degree of Masson’s trichrome stain. *P* < 0.05 compared with different experimental groups. Bars represent mean ± SDM. (*n* = 10).

Examination of liver sections stained with Masson’s trichrome showed that rats received CCl_4_ displayed a significant increase in the amount of collagen fiber deposition in the portal area and around the central veins (Fig. 3B, C). The Masson’s trichrome area (%) was markedly increased in the CCl_4_ treated group (G3) after 4 weeks of experiment (Fig. 3E). On the other hand, treatment of rats with sildenafil and CCl_4_ showed reduced collagen fiber deposition (Fig. 3D). The Masson’s trichrome area (%) was markedly decreased when rats treated with sildenafil and CCl_4_ after 4 weeks of experiment (Fig. 3E). The minimal collagen expression just around the central veins were detected in the control, and sildenafil-treated groups (Fig. 3A).

### Immunohistochemical assessment of liver tissue

**Figure 4:**
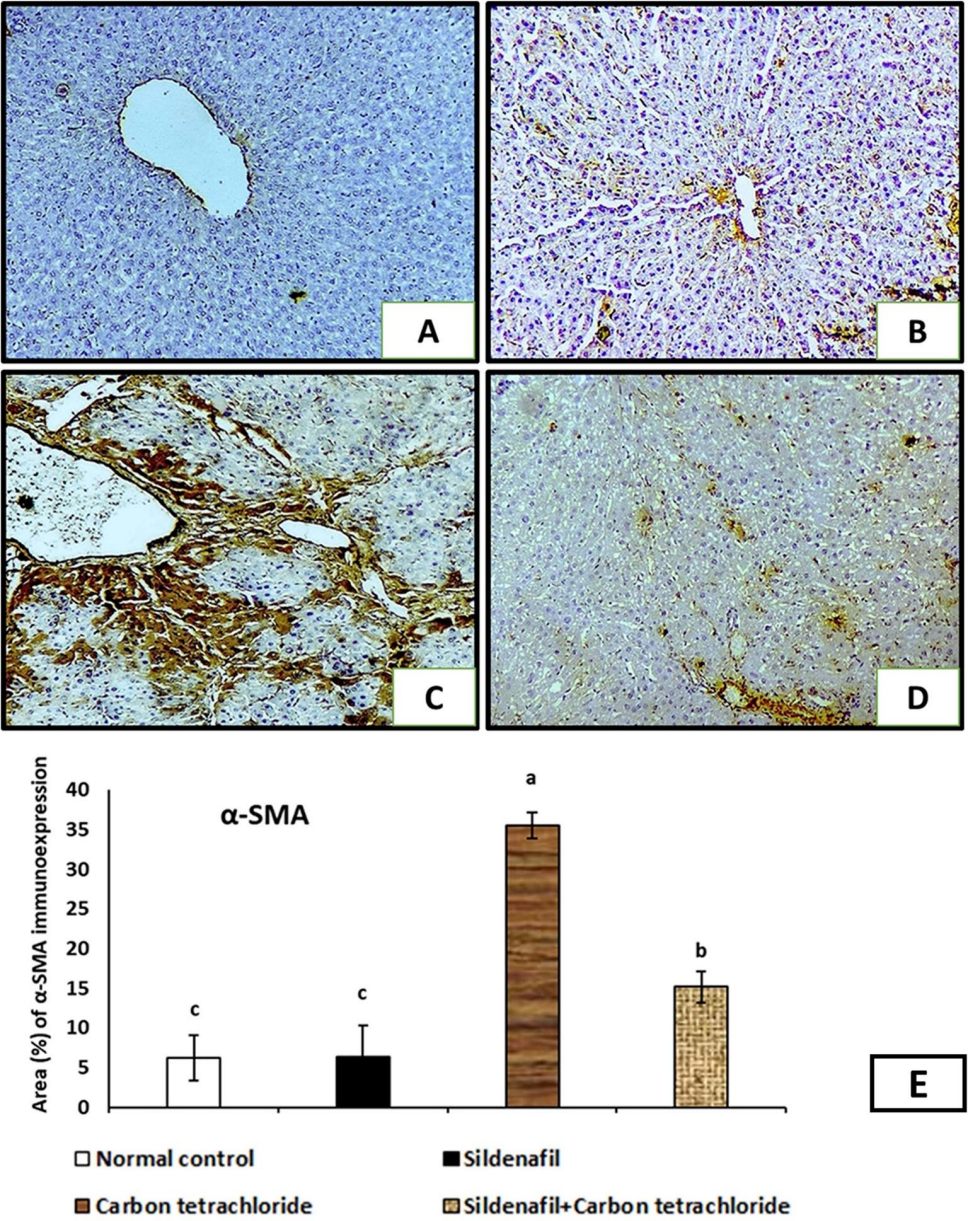
Immunohistochemical expression of α-smooth muscle actin (α-SMA) in liver tissue from different experimental groups. The brown color indicating positivity. (**A**) The control group (G1) showed slight amount of α-SMA-positivity merely seen in vascular wall. (**B**) G2 received sildenafil showed no difference compared with control. (**C**) G3 rats that received CCl_4_ showed the α-SMA protein is highly expressed in the area of central vein, portal tract, and between hepatocytes. (**D**) G4 received sildenafil and CCl_4_ and showed mild α-SMA-expression; however, the level of positivity is lower than in the G3. (Anti-α-SMA immunostaining ×100). (**E**) Digital morphometric study of area (%) of α-SMA immunohistochemical staining sections of liver of experimental rats. Data were used to estimate the degree of α-SMA immunohistochemical staining. *P* < 0.05 compared with different experimental groups. Bars represent mean ± SDM. (*n* = 10).

Α-smooth muscle actin (α-SMA) immunohistochemical staining sections of liver tissues observed that rats exposed to CCl_4_ revealed that α-SMA protein is highly expressed in the area of central vein, portal tract, and between hepatocytes indicating fibrotic changes (Fig. 4C). The α-SMA area (%) was markedly increased in the CCl_4_ treated group (G3) after 4 weeks of experiment (Fig. 4E). While rats treated with sildenafil and CCl_4_ showed mild α-SMA-expression, and the level of positivity is lower than in the CCl_4_ treated rats (Fig. 4D). The α-SMA area (%) was markedly decreased in the sildenafil and CCl_4_ treated group (G4) after 4 weeks of experiment (Fig. 4E). The α-SMA immunohistochemical staining sections of liver tissues of normal control, and sildenafil-treated rats showed a minimal reaction in the vascular wall (Fig. 4A, B).

## Discussion

The purpose of this study was to investigate if sildenafil could adequately support liver function in Wistar rats given CCl_4_ to induce liver fibrosis. Chronic liver diseases causes health problem with high morbidity and mortality rates all over the world^26^. Liver fibrosis, which is defined by an imbalance between excessive matrix synthesis and matrix breakdown, is the result of persistent tissue injury-induced defective wound healing^27^. It has been determined that HSCs are the primary cells that produce matrix along the course of liver fibrosis^28^. Multiple cell types of hepatic progenitor cells are involved in the process of liver repair; the HSCs or liver pericytes, and liver progenitor cells (LPCs) or oval cells are the most important cells involved in this process^29^. Chronic liver injury results in sustained activation of quiescent HSCs (qHPCs) which is transformed into active HPCs (aHPCs) or myofibroblasts, which secrete collagen into the ECM^30^. Furthermore, LPCs are reprogrammed into fiber secreting cells (epithelial to mesenchymal transition) and recruited immune cells (effector–profibrogenic) amplify the process of fibrogenic deposition^31^.

The current study revealed that the activity of ALT, AST and GGT markedly increased that indicated damage of the hepatic tissue of CCl_4_-treated group after 4 weeks administration. This could be the result of inflammation and hepatocellular damage, which raises the permeability of the cell membrane and releases transaminases into the blood stream. A similar results were recorded in rats treated with CCl_4_^32^. GGT activity increase is linked to all types of primary and secondary hepatobiliary diseases; higher blood levels of GGT are associated with cholestasis brought on by intrahepatic or extrahepatic biliary blockage^33^. Marked decreases in serum ALT, AST, and GGT levels in rats co-treated with sildenafil + CCl_4_ were recorded. The hepatoprotective activity may be attributed to an anti-inflammatory property of sildenafil. These findings coincided with Molehin et al.^34^ who found that the sildenafil moderately reduced hepatotoxicity of CCl_4_.

In this work, rats treated with CCl_4_ showed decreased GSH contents and CAT activity after the fourth week of therapy, and an increase in LPO as evidenced by an elevation in MDA levels. This effect may have resulted from damage to cellular components and peroxidation of membrane lipids^35^. The primary mechanism of CCl_4_-induced liver damage is oxidative stress, whereby hepatic cytochrome P450 metabolizes CCl_4_ to produce the trichloromethyl radical; promote hepatocyte necrosis, impair liver functioning, and upset hepatic architecture as a result of free radicals produced by CCl_4_ induction^36^. The reactive oxygen species (ROS) which deplete endogenous antioxidants and interact with cellular lipids leading to lipid-peroxidation^36^. Rats co-treated with sildenafil and CCl_4_ showed decreased LPO (MDA) levels and increased GSH content and CAT activity. These findings corroborate those of Abd El Motteleb et al.^37^, who discovered that sildenafil protects against oxidative stress and liver impairment brought on by bile duct ligation. Sildenafil depresses the generation of hydrogen peroxide by inducing the activity of antioxidants and preventing ROS generation. Thus, sildenafil improved the antioxidants concentrations and reduced oxidative stress. The GSH is the most important cellular antioxidant that plays an important role in scavenging of free radicals^38^.

In the present study, there was statistically significant upregulation of hepatic collagen-1α, IL-1β, OPN, and TGF-β mRNA expression when rats treated with CCl_4_ after the fourth week of the experiment. Collagen-1α, IL-1β, OPN, and TGF-β mRNA expression were all shown to be downregulated in rats given sildenafil and CCl_4_. These findings agree with Kaleta et al.^39^. Sildenafil caused suppressive effects on many pro-inflammatory cytokines through its effect on oxidative and inflammatory pathways and the overexpression of pro-inflammatory cytokines, such as IL-1β, was reduced by sildenafil; these cytokines have synergistic effects that stimulate immune and non-immune cells, as well as other cytokines and adhesion molecules^40^.

The CCl_4_-treated rats significantly increased the expression of OPN. These findings corroborate those of Kaleta et al.^41^. Hepatic OPN expression correlated with inflammatory cell infiltration in the portal area; during CCl_4_ intoxication, OPN facilitated the accumulation of macrophages at sites of injury and consequently enhance hepatic inflammation, hepatic stellate cells, and fibrogenesis^42^. Phosphodiesterase inhibitors elevate cGMP level, which inhibits fibroblast-to-myofibroblast transition that represent an important source of osteopontin^43^. Moreover, Kaleta et al.^39^ demonstrated that sildenafil downregulates OPN gene expression in human peripheral mononuclear cells; that secret OPN during chronic liver injury as patients with primary biliary cirrhosis, autoimmune hepatitis, hepatic cirrhosis, and primary sclerosing cholangitis have all been shown to have elevated hepatic OPN levels. Sildenafil downregulates OPN gene expression, this may refer to the immunomodulatory and anti-inflammatory effects of sildenafil on human immune system cells^44^. Numerous cell types express OPN during the hepatic fibrotic healing process. Hepatocytes and immune cells (macrophages, Kupffer cells) express more OPN mRNA, and OPN also attracts more neutrophils and macrophages to the area. Moreover, hepatic progenitor cells like HSC express OPN significantly while transitioned from Q-HSC to become MF-HSC then OPN stimulate the migration of hepatic progenitor cells towards the site of necrosis in the liver. It has been demonstrated that OPN stimulates HSC migration, proliferation, and epithelial-mesenchymal transition; it also produces factors that activate nearby progenitor cells and cells that produce ECM. It contributes to liver tissue scarring in both an autocrine and paracrine manner^45^.

The CCl_4_-treated rats significantly increased the expression of hepatic collagen-1α and TGF-β. While co-treated with sildenafil + CCl_4_ showed down regulation of these genes. These findings corroborate those of Abd El Motteleb et al.^37^. A profibrogenic reaction was set off by damaged liver cells, which increased TGF-β expression and transformed latent TGF-β into active form, which activated HSCs. Higher hepatic expression of collagen-1α indicates upregulation of TGF-β gene content linked to HSC activation. TGF-β1 phosphorylates Smad2/3 proteins via activating its own receptors on HSCs. Increased production of collagen-1α is caused by phosphorylated Smad2/3 protein translocating into the nucleus and forming a complex with Smad4. One important cytokine that has been identified as mediating liver fibrosis is TGF-β^46^. Activated HSCs promote the migration and proliferation of myogenic markers, such as α-SMA, transforming them into the primary cells that produce collagen-1α; the primary constituent of the ECM that accumulates and plays a crucial role in the onset and progression of liver fibrosis^47^. The group that received sildenafil and CCl_4_ had lower levels of hepatic TGF-β and collagen-1α gene expression; these findings are consistent with those reported by Xiang et al.^48^. The potential reason for sildenafil’s antifibrotic impact could be its ability to inactivate HSC through the inhibition of TGF-β production^49^.

The improvement in α-SMA immunohistochemistry characteristics of the liver of rats treated with CCl_4_ was corroborated by these data; revealed that α-SMA protein is highly expressed in the area of central vein, portal tract, and between hepatocytes indicating fibrotic changes^50^. While rats treated with sildenafil and CCl_4_ showed mild α-SMA-expression. α-SMA is frequently employed as a diagnostic tool for the initial phases of fibrosis^51^.

These findings were consistent with the improvement in the liver’s histological characteristics of sildenafil-treated rats compared with the liver of CCl_4_-treated rats, which showed sever degenerative changes, vacuolization, sever congested central vein, congested portal tract vasculature, large inflammatory cells infiltrates, in addition to congested and dilated sinusoids. The outcomes aligned with the results of an earlier investigation^37^ that used bile duct ligation to cause liver fibrosis and sildenafil co-treatment. These findings were also corroborated by those of the Masson’s trichrome stain examination. It revealed the improvement of collagen fiber deposition in the portal area and around the central veins in rats treated with sildenafil and CCl_4_. These results aligned with those of an earlier investigation^52^ that induced liver fibrosis in rats by CCl_4_ and co-treated with Zataria multiflora Boiss essential oil.

## Conclusion

Oxidative stress and profibrotic genes (OPN and TGF-β) played pivotal role in chronic liver diseases. Modulation of oxidative stress and suppression of profibrotic genes by sildenafil decreased hepatic progenitor response and protected the liver against fibrosis. Thus, sildenafil is a potentially attractive anti-fibrotic strategy in the liver fibrosis.

### Supplementary Information

Supplementary Information.

## Data Availability

The corresponding author will deliver the information needed to back up this study's conclusions upon reasonable request and with permission from the Department of Forensic Medicine and Toxicology, Faculty of Veterinary Medicine, Benha University. The protocol number for approval is: BUFVTM 09-11-22.
