# Supplementary material for: Ameliorative effects of sildenafil against carbon tetrachloride induced hepatic fibrosis in rat model through downregulation of osteopontin gene expression
Source: Sci Rep. 2024 Jul 23;14:16902. doi: 10.1038/s41598-024-67305-1 (PMC11266717; doi:10.1038/s41598-024-67305-1)
Supplement: Supplementary file 1 — Supplementary Information. [file 41598_2024_67305_MOESM1_ESM.docx]

**Research article**

**Ameliorative effects of sildenafil against carbon tetrachloride induced hepatic fibrosis in rat model: a possible role of osteopontin.**

**Highlights:**

1- Sildenafil reduces ROS production that decreased hepatotoxicity induced by carbon tetrachloride.

2- Sildenafil down-regulates the mRNA expressions of profibrotic genes [collagen-1α, IL-1β, osteopontin (OPN), and TGF-β].

3- Sildenafil is a potentially attractive anti-fibrotic strategy in the liver fibrosis.
